# LCZ696, an Angiotensin Receptor-Neprilysin Inhibitor, Improves Cardiac Hypertrophy and Fibrosis and Cardiac Lymphatic Remodeling in Transverse Aortic Constriction Model Mice

**DOI:** 10.1155/2020/7256862

**Published:** 2020-01-11

**Authors:** Qing Ge, Li Zhao, Chen Liu, Xiaoming Ren, Yi-hui Yu, Chang Pan, Zuoying Hu

**Affiliations:** Department of Cardiology, Nanjing First Hospital, Nanjing Medical University, No. 68, Changle Road, Nanjing 210006, Jiangsu, China

## Abstract

Cardiac hypertrophy and ventricular remodeling following heart failure are important causes of high mortality in heart disease patients. The cardiac lymphatic system has been associated with limited research, but it plays an important role in the improvement of myocardial edema and the promotion of fluid balance. LCZ696 is a novel combination of angiotensin and neprilysin inhibitors. Here, we studied the role played by LCZ696 during transverse aortic constriction (TAC) induced cardiac hypertrophy and changes in the lymphatic system. Mice undergoing aortic coarctation were constructed to represent a cardiac hypertrophy model and then divided into random groups that either received treatment with LCZ696 (60 mg/kg/d) or no treatment. Cardiac ultrasonography was used to detect cardiac function, and hematoxylin and eosin (H&E) and Masson staining were used to detect myocardial hypertrophy and fibrosis. The proinflammatory factors interleukin-6 (IL-6), IL-1*β*, and tumor necrosis factor-*α* (TNF-*α*) were detected in the blood and heart tissues of mice. The protein expression levels of lymphatic-specific markers, such as vascular endothelial growth factor C (VEGF-C), VEGF receptor 3 (VEGFR3), and lymphatic vessel endothelial hyaluronan receptor 1 (LYVE-1) were detected in mouse heart tissues. We also examined the colocalization of lymphatic vessels and macrophages by immunofluorescence. The results showed that LCZ696 significantly improved heart dysfunction, cardiac hypertrophy, and fibrosis and inhibited the expression of proinflammatory factors IL-6, IL-1*β*, and TNF-*α* in the circulating blood and heart tissues of mice. LCZ696 also decreased the protein expression levels of VEGF-C, VEGFR3, and LYVE-1 in mouse heart tissues, ameliorated the transport load of lymphatic vessels to macrophages, and improved the remodeling of the lymphatic system in the hypertrophic cardiomyopathy model induced by TAC.

## 1. Introduction

Heart failure remains one of the leading causes of mortality and morbidity worldwide, despite great improvements in treatments for associated diseases. Previous studies have demonstrated that LCZ696, which is an angiotensin receptor-neprilysin inhibitor (ARNI), improved cardiac function, with the attenuation of fibrosis, when used to treat several types of heart failure with reduced ejection fractions [1]. In the PARAMOUNT study (a prospective comparison between an ARNI and an angiotensin receptor blocker (ARB) for the management of heart failure with preserved ejection fraction (HFpEF)), LCZ696 treatment reduced the serum levels of the N-terminal, pro-B-type, natriuretic peptide, compared with valsartan treatment after 12 weeks, and was well-tolerated when used for the treatment of HFpEF [2]. LCZ696 attenuated cardiac remodeling and dysfunction after myocardial infarction (MI) by reducing cardiac fibrosis and hypertrophy [3]. Moreover, LCZ696 treatment significantly ameliorated cardiac hypertrophy, inflammation, and vascular endothelial dysfunction in high-salt loaded spontaneously hypertensive rats compared with valsartan treatment [4]. Furthermore, LCZ696 treatment inhibited cardiac hypertrophy, fibrosis, and vasculopathy in a rat model of chronic kidney disease [5]. However, LCZ696 treatment improved isoproterenol-induced cardiac fibrosis, but not hypertrophy, in rats [6]. The effects of LCZ696 on pressure overload-induced cardiac hypertrophy remain unclear, and the possible potential mechanisms are unknown.

The heart has an extensive lymphatic network that regulates and maintains fluid balance [7]. The cardiac lymphatic system has become an active target for research, and recent advances in this field have provided new insights into the treatment of cardiovascular diseases. The ischemic heart exhibits a dysfunctional lymphatic network that participates in the development of chronic myocardial edema and aggravates cardiac dysfunction [8]. Lymphangiogenic therapy has also been successfully used to resolve edema formation, inflammatory cell accumulation, and fibrosis in MI mice [8]. However, sustained lymphangiogenesis can increase the exposure of lymph node targets, which can modulate adverse immune reactions [9]. Mouse studies using transplanted hearts that carry a lymphatic endothelial cell- (LEC-) specific vascular endothelial growth factor receptor 3 (VEGFR3) deletion confirmed that VEGFR3 inhibition leads to prolonged cardiac allograft survival [10]. How the cardiac lymphatic system changes during stress overload-induced cardiac hypertrophy and after LZC696 administration remains unknown.

To shed light on the potential effects of LCZ696 on hypertrophy and fibrosis in transverse aortic constriction (TAC) model mice, we applied histological and quantitative polymerase chain reaction (q-PCR) analyses to TAC hypertrophy model mouse hearts. By measuring the levels of VEGF-C, VEGFR3, and lymphatic vessel endothelial hyaluronan receptor 1 (LYVE-1), using western blots, and examining the colocalization of lymphatic vessels and macrophages, using immunofluorescence, we explored the changes that occurred in the cardiac lymphatic system and the role played by LCZ696 in TAC mice.

## 2. Materials and Methods

### 2.1. Animal Experiment

Adult, male, C57BL/6 mice (8–10 weeks old, 22–24 g) were purchased from Nanjing Medical University Animal Laboratory and were housed at 20–24°C, under a 12 h light-dark cycle, at the Laboratory Animal Centre of Nanjing First Hospital. Food and water were freely available throughout the experiment. The study protocol was approved by the Animal Care Committees of the Laboratory Animal Centre & Nanjing First Hospital. Mice were randomly assigned to one of the following five groups: sham, TAC 1 week, TAC + LCZ696 1 week, TAC 4 weeks, and TAC + LCZ696 4 weeks. As reported previously [11, 12], TAC was performed to establish a pressure overload-induced cardiac hypertrophy model. Briefly, C57BL/6 mice were anesthetized with 2.0% isoflurane, placed on a heated surgical board, and given subcutaneous 2.5 mg/kg flunixin. Then, the chest was entered, and the aortic arch was isolated. TAC was performed using a 6-0 suture, which was tied around a 27-gauge needle and the aortic arch. Then, the needle was removed to induce 60%–80% constriction of the aorta. A sham surgical operation was performed for the sham group, in which the transverse aorta was exposed but was not constricted. The survival rate of mice after TAC surgery was 68%. To test whether LCZ696 could inhibit cardiac hypertrophy, we treated the surviving mice with either saline (sham group, TAC group) or LCZ696, at 60 mg/kg/day (TAC + LCZ696 group), starting on the second day after surgery. The LCZ696 dose was based on previous reports [13, 14]. Four weeks later, after echocardiography analyses of the mice were performed, all animals were sacrificed, and cardiac tissues were stored for further studies.

### 2.2. Echocardiography

The cardiac structures and functions of the mice were measured using the Vevo2100 system (Fujifilm Visual Sonics, Toronto, Canada), with a high-frequency (30 MHz) MS‐400 transducer, at the Animal Centre of Nanjing Medical University. Mice were anesthetized by inhalation of isoflurane prior to the cardiac ultrasound. The isoflurane inducing dose was 3%, whereas the maintenance dose was 1.5%. The cardiac functions of the mice were assessed by measuring left ventricular internal dimension in systole (LVIDS), left ventricular internal dimension in diastole (LVIDD), left ventricular systolic posterior wall thickness (LVPWS), left ventricular diastolic posterior wall thickness (LVPWD), interventricular septal end-systolic thickness (IVSS), interventricular septal end-diastolic thickness (IVSD), left ventricular ejection fraction (EF), short-axis fraction (FS), and corrected left ventricular mass index (LV Mass Corrected).

### 2.3. Western Blots

To measure the levels of interleukin (IL)-6, IL-1*β*, tumor necrosis factor-*α* (TNF-*α*), VEGF-C, VEGFR3, and LYVE-1, equal amounts of the samples were loaded onto 10% sodium dodecyl sulfate (SDS)-polyacrylamide gels. Then, the resolved proteins were transferred to a polyvinylidene difluoride (PVDF) membrane and incubated for one hour, at room temperature, in blocking solution (5% nonfat dried milk dissolved in Tris-buffered saline with Tween 20 [TBST] buffer). Then, the filters were probed, overnight, at 4°C, in blocking solution containing primary antibodies, diluted 1 : 1,000, against the following: IL-6 (MB9296, Bioworld), IL-1*β* (BS6067, Bioworld), TNF-*α* (11948S, Cell Signaling Technology), VEGF-C (sc-374628, Santa Cruz Biotechnology), VEGFR3 (ab27278, Abcam), LYVE-1 (ab14917, Abcam), and *β*-actin (AP0060, Bioworld). Membranes were washed twice with TBST buffer and incubated with horseradish peroxidase-conjugated secondary antibody (Cell Signaling Technology), for 1 hour, at room temperature, followed by washing three times. Signal detection was performed using an enhanced chemiluminescence substrate (Millipore, USA) and quantitated using Image J software.

### 2.4. RNA Quantitative Reverse Transcriptase-Polymerase Chain Reaction Analysis (qRT-PCR)

Total RNA was extracted from cardiac tissue using TRIzol reagent (Invitrogen, USA). cDNA was produced using the PrimeScript RT reagent kit (TakaRa Biotechnology, China). qRT-PCR was performed on an ABI 7500 system (Grand Island, NY, USA), using the SYBR Premix Ex Taq kit (TakaRa Biotechnology, China). The reaction conditions were as follows: 95°C for 30 s for predenaturation, followed by 40 cycles of 95°C for 5 s and 60°C for 34 s. Glyceraldehyde 3-phosphate dehydrogenase (GAPDH) was used as a reference gene. All primers used are listed in Table 1.

### 2.5. Histological Analysis

Cardiac tissues isolated from mice were fixed in 10% formalin, overnight, at room temperature, dehydrated through increasing concentrations of ethanol, and then embedded in paraffin. Then, the samples were cut into 5 *μ*m sections and stained with hematoxylin and eosin (H&E) to analyze the heart morphology. The samples were also stained with Masson trichrome to analyze the extent of myocardial fibrosis [15]. The results were observed using an Olympus-BHS microscope (San Jose, CA), attached to a QImaging Retiga 4000RV digital camera (Surrey, British Columbia, Canada), and measured with Image J software.

### 2.6. Immunofluorescence Staining

Frozen heart tissue sections were immunolabeled, using a lymphatic marker (LYVE-1) and a macrophage marker (CD68), to observe cardiac lymphangiogenesis and the colocalization between lymphatic vessels and macrophages [8, 16]. The tissue was fixed with 4% paraformaldehyde, for 20 minutes, at room temperature, then permeabilized with 0.5% Triton X-100 for 20 minutes. After blocking with 5% bovine serum albumin (BSA), for 1 hour, at room temperature, the tissues were incubated with a primary LYVE-1 antibody (ab14917, Abcam) and a CD68 antibody (ab53444, Abcam), at 4°C, overnight. 4′-6-Diamidino-2-phenylindole (DAPI, Sigma) was used as a nuclear marker. Finally, fluorescent images were obtained using a laser confocal microscope.

### 2.7. Expression of Proinflammatory Cytokines

The expression levels of the proinflammatory factors IL-6, IL-1*β*, and TNF-*α* in mouse serum were detected using corresponding enzyme-linked immunosorbent assay (ELISA) kits (R&D Systems, Minnesota, MN, USA).

### 2.8. Statistical Analysis

All data were analyzed using SPSS 22.0 (IBM Corporation, Armonk, USA). Data are presented as the mean ± standard deviation. The Shapiro–Wilk test was used to examine the normality of all variables. For normally distributed data, an unpaired Student's *t*-test was used for binary comparisons, whereas a one-way analysis of variance (ANOVA) was employed for multiple comparisons. For skewed data distributions, the differences among variables were analyzed using the Wilcoxon signed-rank or the Friedman test. *P* < 0.05 was considered to be statistically significant.

## 3. Results

### 3.1. LCZ696 Ameliorated Cardiac Function in TAC Model Mice

**Figure 1:**
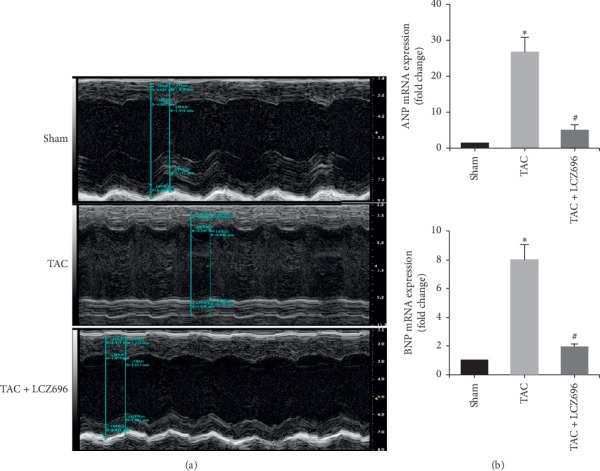
LCZ696 improved heart dysfunction in TAC mice. (a) Echocardiography was performed in mice treated with or without LCZ696, at the indicated dose. (b) The mRNA expression levels of ANP and BNP were measured by RT-PCR. Data in (a) and (b) were obtained from six mice in each group. All data are expressed as the mean ± SD. ^*∗*^*P* < 0.05 vs. sham; ^#^*P* < 0.05 vs. TAC.

TAC was applied to induce cardiac hypertrophy in mice, and the development of cardiac hypertrophy and the regression of the established cardiac hypertrophy were confirmed by echocardiography analyses. After surgery, the TAC mice were randomized into either a group that received treatment with LCZ696 (60 mg/kg) for 4 weeks or a group that received no treatment. As shown in Figure 1(a), the cardiac function was significantly impaired in the TAC alone group but was ameliorated in the LCZ696 treatment group. In addition, heart function indicators, such as LVIDS, LVIDD, LVPWS, LVPWD, IVSS, IVSD, EF%, and FS%, were significantly improved in the LCZ696 treatment group compared with the untreated group (Table 2). Significant differences were also observed for the index of left ventricular mass between these groups (Table 2).

The expression levels of atrial natriuretic peptide (ANP) and brain natriuretic peptide (BNP) mRNA in the left ventricle were also examined by q-PCR and were significantly increased in the TAC group compared with the control group but decreased in the LCZ696 treatment group, which indicated that LCZ696 was able to improve heart failure induced by pressure overload (Figure 1(b)).

### 3.2. LCZ696 Alleviated Cardiac Hypertrophy and Fibrosis in TAC Mice

**Figure 2:**
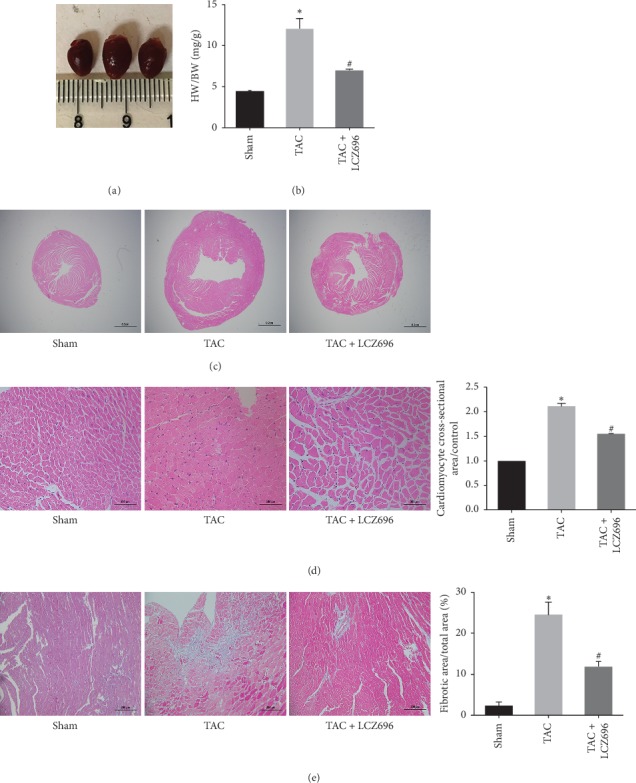
LCZ696 inhibited cardiac hypertrophy and fibrosis. (a) The morphologies of mouse hearts, from left to right: sham group, TAC group, TAC + LCZ696 group. (b) The ratio of heart weight to body weight (HW/BW). (c, d) The cardiac morphologies and cardiomyocytes were analyzed by hematoxylin and eosin (H&E) staining, separately, and examined under 20x and 400x magnification. (e) Cardiac fibrosis was measured by Masson trichrome staining and examined under 200x magnification. Data in (a)–(e) were obtained from six mice in each group. All data are expressed as the mean ± SD. ^*∗*^*P* < 0.05 vs. sham; ^#^*P* < 0.05 vs. TAC.

As shown in Figures 2(a) and 2(b), compared with the sham group, the left ventricles in the TAC group were hypertrophic, with increased left ventricular cavities. The cardiomyocytes were enlarged in the TAC group, based on H&E staining viewed under a 2x objective, whereas LCZ696 was able to significantly alleviate cardiac hypertrophy and improve the ventricular remodeling caused by TAC in mice (Figure 2(c)). The cross-sectional area of cardiomyocytes in the TAC group was also larger than that in the sham group but was smaller in the LCZ696 group, based on H&E staining viewed under a 40x objective (Figure 2(d)). These results further indicated that LCZ696 treatment was able to inhibit cardiac hypertrophy and improve ventricular remodeling.

Hypertrophy and cardiomyocyte apoptosis resulted in the deposition of collagen fibers and fibrosis of the heart. As shown in Figure 2(e), the collagen fibers were stained blue by Masson staining and myocardial fibrosis in the TAC group was significantly increased compared with that in the sham group but was decreased in the LCZ696 treatment group. These results demonstrated that LCZ696 treatment could inhibit fibrosis induced by pressure overload.

### 3.3. LCZ696 Treatment Inhibited the Inflammatory Response in TAC Mice

**Figure 3:**
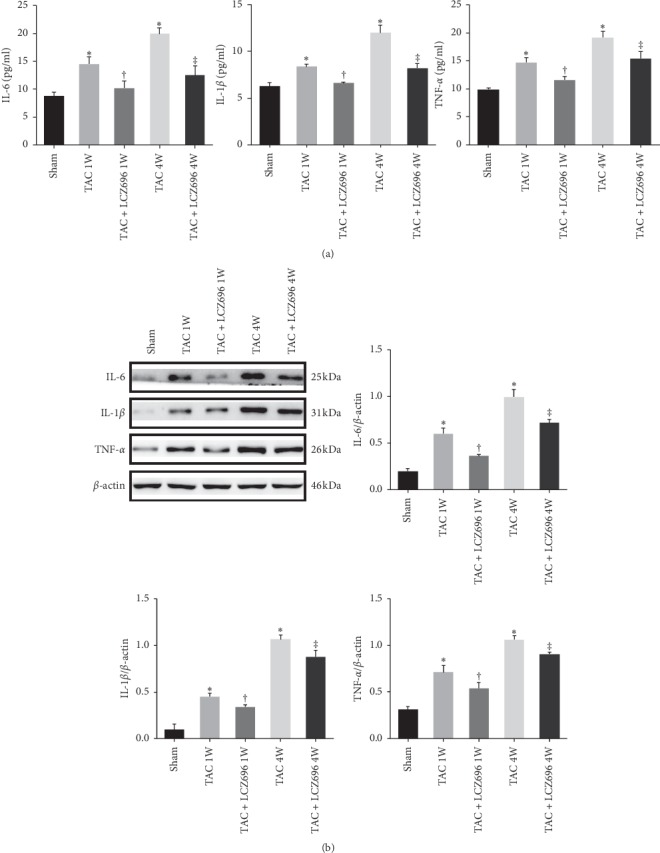
LCZ696 inhibits the expression of proinflammatory factors in circulating blood and heart tissues. (a) The expression levels of the proinflammatory factors IL-6, IL-1*β*, and TNF-*α* in the circulating blood of mice were measured by enzyme-linked immunosorbent assay. (b) The protein expression levels of IL-6, IL-1*β*, and TNF-*α* in mouse heart tissues were detected by western blots. Data in (a) were obtained from six mice in each group. Data in (b) were obtained from four mice in each group. All data are expressed as the mean ± SD. ^*∗*^*P* < 0.05 vs. sham; ^†^*P* < 0.05 vs. TAC 1W; ^‡^*P* < 0.05 vs. TAC 4W. 1W indicates 1 week, 4W indicates 4 weeks.

The inflammatory response is an important factor in accelerating the development of chronic heart failure. To better understand changes in the expression of inflammatory factors that occur in TAC mice and the effects of LCZ696 treatment on the timing of these changes, we also examined TAC mice after 1 week, either with or without LCZ696 treatment. As shown in Figure 3(a), the expression levels of the proinflammatory factors IL-6, IL-1*β*, and TNF-*α* in the circulating blood of TAC mice after 1 week were only slightly increased compared with those of the sham group, whereas these levels were significantly increased in TAC mice after 4 weeks. The administration of LCZ696 for both 1 week and 4 weeks was able to reduce the expression levels of proinflammatory factors compared with the respective TAC groups. This result is consistent with the protein expression levels observed for proinflammatory factors in mouse heart tissues (Figure 3(b)).

### 3.4. LCZ696 Treatment Reduced the Transport Loads of Lymphatic Vessels to Macrophages and Improved the Remodeling of the Lymphatic System in TAC Mice

**Figure 4:**
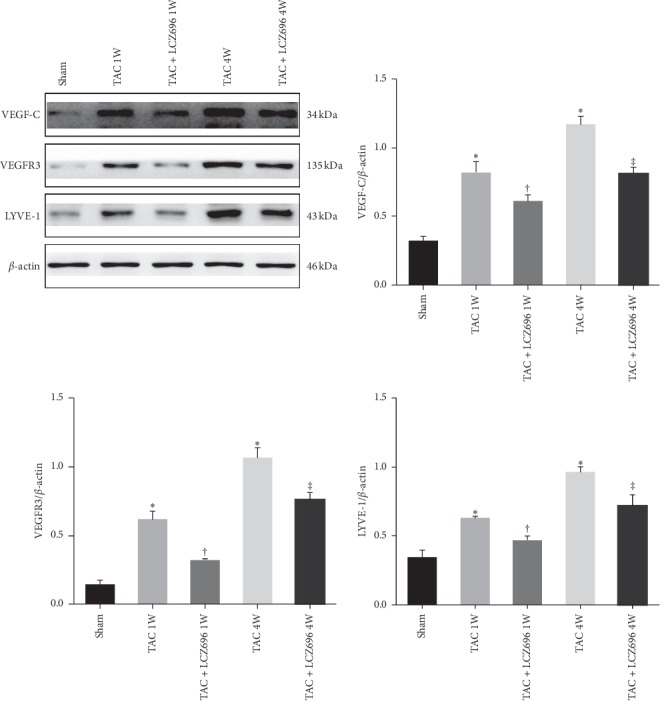
LCZ696 altered the expression of lymphatic endothelial markers. The protein expression levels of VEGF-C, VEGFR3, and LYVE-1 were determined by western blot. Data were obtained from four mice in each group. All data are expressed as the mean ± SD. ^*∗*^*P* < 0.05 vs. sham; ^†^*P* < 0.05 vs. TAC 1W; ^‡^*P* < 0.05 vs. TAC 4W. 1W indicates 1 week, 4W indicates 4 weeks.

**Figure 5:**
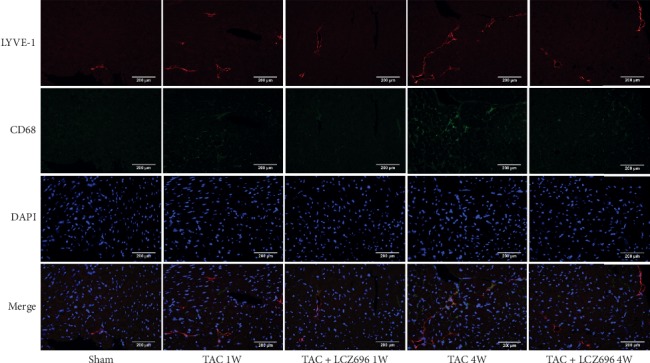
LCZ696 improved the remodeling of the lymphatic system and the accumulation of CD68 macrophages in mouse heart tissues, as assessed by immunofluorescence, under 400x magnification. Data were obtained from four mice in each group. 1W indicates 1 week, 4W indicates 4 weeks.

Myocardial lymphangiogenesis has been observed during heart failure in the MI model. To explore the potential mechanism through which LCZ696 treatment improves myocardial hypertrophy, we further evaluated myocardial lymphangiogenesis by detecting the expression of regulatory factors and lymphatic markers. The expression levels of VEGF-C, VEGFR3, and LYVE-1 were detected by western blot. As shown in Figure 4, the protein expression levels of VEGF-C, VEGFR3, and LYVE-1 in the heart tissues of mice began to increase in the first week after TAC surgery, suggesting an increase in lymphangiogenesis, and reached a higher level by the fourth week after surgery; however, these levels were decreased by the administration with LCZ696 for 1 and 4 weeks. In addition, immunofluorescence staining for LYVE-1 and CD68 showed that the number of lymphatic vessels increased in mice 1 week after TAC, accompanied by the aggregation of macrophages around lymphatic vessels (Figure 5). This phenomenon was even more obvious 4 weeks after TAC. However, the number of lymphatic vessels in the heart tissues of mice treated with LCZ696 for both 1 week and 4 weeks was reduced compared with the corresponding TAC groups, and the observed accumulation of macrophages around the lymphatic vessels was also reduced.

## 4. Discussion

In our study, aortic coarctation caused the increased expression of proinflammatory factors in the circulating blood and heart tissues of mice and increased lymphangiogenesis and the accumulation of macrophages near the lymphatic vessels. These effects were significantly ameliorated by LCZ696 treatment, regardless of the treatment length. In addition, cardiac edema, fibrosis, and dysfunction also improved in TAC mice treated with LCZ696. Therefore, exploring the potential mechanisms through which LCZ696 treatment improves the symptoms of TAC could have important clinical implications.

Inflammation, followed by fibrosis, is an important pathological feature of pathological hypertrophy [17]. In our experiments, the observed increase in proinflammatory factor expression levels in both the blood and heart tissues of TAC mice and the accumulation of macrophages demonstrated that the inflammatory response is active in the TAC model. However, treatment with LCZ696 was able to significantly inhibit the inflammatory response induced by TAC, which is consistent with our previous findings in a model of diabetic cardiomyopathy [18]. These data all suggested that LCZ696 treatment suppresses inflammation, which is a potential trigger of cardiac fibrosis.

LCZ696, a combination of the angiotensin II receptor blocker valsartan and the neprilysin inhibitor sacubitril, has been shown to improve cardiac functions in previous studies; however, the underlying mechanisms through which LCZ696 acts remain unclear. LCZ696 has been reported to improve cardiac function, with the attenuation of fibrosis, through the transforming growth factor-*β* (TGF-*β*) signaling pathway in a streptozotocin-induced diabetic mouse model of heart failure with reduced ejection fraction [13]. von Lueder reported that LCZ696 attenuated cardiac remodeling and dysfunction after MI by reducing cardiac fibrosis and hypertrophy [3]. In addition, LCZ696 was shown to improve cardiac function by alleviating dynamin-related protein 1- (Drp1-) mediated mitochondrial dysfunction in a mouse model of doxorubicin-induced dilated cardiomyopathy [15]. In our study, we found that cardiac hypertrophy and myocardial fibrosis were significantly inhibited in TAC mice by LCZ696 treatment. LCZ696 further improved cardiac hypertrophy in this TAC model, which represents another type of heart failure.

The heart has a broad lymphatic network that regulates the fluid balance in the interstitial tissue, and dysfunction of the lymphatic system can lead to myocardial edema. Many cardiovascular diseases, such as MI and chronic heart failure, have been associated with myocardial edema. During myocardial edema, many inflammatory cytokines are expressed and oxygen free radicals are generated, which have negative effects on lymphatic function, leading to dysplasia of lymphatic flow, lymphedema, and chronic inflammation [19, 20]. Our study suggested that the inhibitory effects of LCZ696 treatment on TAC-induced cardiac lymphatic vessel remodeling may be attributed to the inhibition of inflammatory cytokine expression, which in turn, reduced the lymphatic transport load to immune cells. Lymphatic endothelial markers, such as VEGFR3, LYVE-1, podoplanin, and Prox-1, have been confirmed to play key roles in the development of the lymphatic system and have been widely used for the detection of lymphangiogenesis and lymphatic vessel invasion [21–23]. In our study, the numbers of myocardial LYVE-1-labeled lymphatic vessels and CD68 macrophages began to increase one week after TAC and continued to increase through four weeks after TAC; however, both were reduced by LCZ696 treatment. In addition, the changes in protein expression levels observed for VEGF-C, VEGFR3, and LYVE-1 in heart tissues of mice, as measured by western blots, were consistent with the immunofluorescence results. Myocardial edema, stimulated by inflammatory factors during cardiac hypertrophy, may lead to lymph flow dysplasia, abnormal lymphatic genesis, and the increased expression of LYVE-1. Treatment with LCZ696 was able to inhibit the expression of inflammatory factors and reduce the degree of myocardial edema, which may be related to reduced lymphangiogenesis and decreased LYVE-1 expression.

A recent study has shown that the selective stimulation of cardiac lymphangiogenesis can improve cardiac dysfunction caused by acute MI [8]. However, the increase in lymphatic vessels caused by acute MI was composed primarily of capillary lymphatic vessels, with diameters of less than 5 *μ*m, which have no transport effects, whereas the number of collecting lymphatic vessels with transport function was reduced. Therefore, to better understand the impacts of LCZ696 treatment on the lymphatic system in the TAC model, further research is necessary to distinguish changes in capillary lymphatic vessels from changes in collecting lymphatic vessels. Interestingly, another study has reported that the inhibition of lymphangiogenesis by the VEGFR3 inhibitor SAR131675 improved renal function in a type 2 diabetic mouse model [24]. Whether the effects of VEGFR3 agonists or inhibitors on the lymphatic system are due to changes in the numbers, transport functions, or conformations of lymphatic vessels remains unknown. Therefore, whether LCZ696 treatment has direct effects on the lymphatic system, as is observed for VEGFR3 agonists or inhibitors, requires further exploration. The use of VEGFR3 agonists or inhibitors has not yet been reported in the TAC model. In the future, we will conduct further research in this area to explore the role played by lymph in the cardiovascular system and to examine potential novel therapeutic strategies for treating heart failure.

## 5. Conclusions

LCZ696 treatment improved cardiac hypertrophy, fibrosis, and cardiac lymphatic remodeling by inhibiting the inflammatory response in TAC model mice.

## Figures and Tables

**Table 1:** Sequence of the internal reference and the paired primers.

	5′-3′ (sense)	5′-3′ (antisense)
GAPDH	ACAGCAACAGGGTGGTGGAC	TTTGAGGGTGCAGCGAACTT
ANP	TGAGTGAGCAGACTGAGGAA	TGGATCTTCGTAGGCTCCGA
BNP	ACAGAAGCTGCTGGAGCTGA	CCGATCCGGTCTATCTTGTG

**Table 2:** Characteristics of echocardiography in experimental mice.

	Sham	TAC	TAC + LCZ696 (60 mg/kg/d)
LVIDS (mm)	2.73 ± 0.17	3.80 ± 0.61^*∗*^	3.07 ± 0.25^#^
LVIDD (mm)	3.98 ± 0.23	4.69 ± 0.45^*∗*^	4.02 ± 0.22^#^
LVPWS (mm)	1.20 ± 0.06	1.35 ± 0.15^*∗*^	1.19 ± 0.12^#^
LVPWD (mm)	0.72 ± 0.03	1.02 ± 0.13^*∗*^	0.89 ± 0.05^#^
IVSS (mm)	1.12 ± 0.07	1.41 ± 0.15^*∗*^	1.21 ± 0.08^#^
IVSD (mm)	0.69 ± 0.02	1.03 ± 0.12^*∗*^	0.88 ± 0.05^#^
EF (%)	59.74 ± 1.78	38.17 ± 8.22^*∗*^	48.27 ± 4.35^#^
FS (%)	31.33 ± 1.21	18.53 ± 4.31^*∗*^	24.01 ± 2.55^#^
LV mass corrected (mg)	79.30 ± 8.02	173.47 ± 45.72^*∗*^	109.65 ± 8.28^#^

*Notes.* LVIDS, left ventricular internal dimension in systole; LVIDD, left ventricular internal dimension in diastole; LVPWS, left ventricular systolic posterior wall thickness; LVPWD, left ventricular diastolic posterior wall thickness; IVSS, interventricular septal end-systolic thickness; IVSD, interventricular septal end-diastolic thickness; EF, left ventricular ejection fraction; FS, short-axis fraction; LV mass corrected, corrected left ventricular mass index. Data shown were obtained from eight mice in each group. Data are shown as the mean ± SD. ^*∗*^*P* < 0.05 versus the sham group; ^#^*P* < 0.05 versus the TAC group.

## Data Availability

The data used to support the findings of this study are available from the corresponding author upon request.
